# Further insights from structural mass spectrometry into endocytosis adaptor protein assemblies

**DOI:** 10.1016/j.ijms.2019.116240

**Published:** 2020-01

**Authors:** Johannes Heidemann, Knut Kölbel, Albert Konijnenberg, Jeroen Van Dyck, Maria Garcia-Alai, Rob Meijers, Frank Sobott, Charlotte Uetrecht

**Affiliations:** a Heinrich Pette Institute, Leibniz Institute for Experimental Virology, Martinistrasse 52, 20251, Hamburg, Germany; b University of Antwerp, Biomolecular & Analytical Mass Spectrometry, Chemistry Dept. Campus Groenenborger V4, Groenenborgerlaan, 171 2020, Antwerp, Belgium; c European Molecular Biology Laboratory (EMBL), Hamburg Outstation, Notkestrasse 85, 22607, Hamburg, Germany; d Astbury Centre for Structural Molecular and Cellular Biology, School of Molecular and Cellular Biology, University of Leeds, LS3 9JT, United Kingdom; e European XFEL GmbH, Holzkoppel 4, 22869, Schenefeld, Germany

**Keywords:** ANTH/Sla2, Epsin, ENTH, Native mass spectrometry, Ion mobility, Surface induced dissociation, CME, Clathrin-mediated endocytosis, ANTH, AP 180 NT homology domains, ENTH, Epsin NT homology domain, PIP2, phosphatidylinositol-(4,5)-bisphosphate, MS, mass spectrometry, SAXS, small angle X-ray scattering, EM, electron cryo microscopy, PA, projection approximation algorithm, CID, collision-induced dissociation, SID, surface-induced dissociation, IM, ion mobility, ^TW^CCS_N2_ / CCS′, collision cross sections, ATD, arrival time distribution, GDH, glutamate dehydrogenase

## Abstract

As a fundament in many biologically relevant processes, endocytosis in its different guises has been arousing interest for decades and still does so. This is true for the actual transport and its initiation alike. In clathrin-mediated endocytosis, a comparatively well understood endocytic pathway, a set of adaptor proteins bind specific lipids in the plasma membrane, subsequently assemble and thus form a crucial bridge from clathrin to actin for the ongoing process. These adaptor proteins are highly interesting themselves and the subject of this manuscript. Using many of the instruments that are available now in the mass spectrometry toolbox, we added some facets to the picture of how these minimal assemblies may look, how they form, and what influences the structure. Especially, lipids in the adaptor protein complexes result in reduced charging of a normal sized complex due to their specific binding position. The results further support our structural model of a double ring structure with interfacial lipids.

## Highlights

• IM-MS and SID reveals that reduced charge states are a result of interfacial lipids.
• ANTH subunits in the outer ring interact barely with each other and stabilize ENTH interaction.
• Additional intermediates confirm our recent double ring model.

## Graphical abstract

**Figure:**
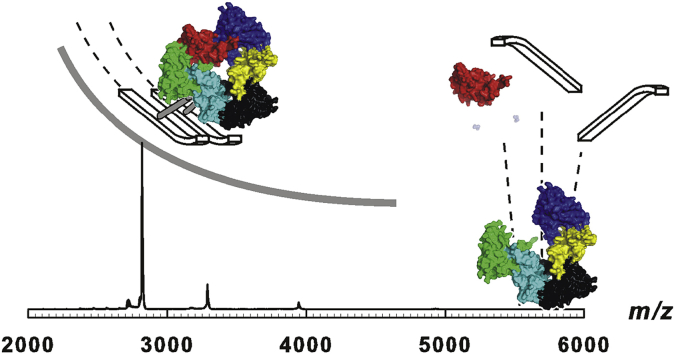

## 1 Introduction

One of the ultimate keys to life, as we know it today, is compartmentalization by physical, membranous barriers enclosing reaction rooms [1]; another one is overcoming exactly these boundaries [2]. Transport across membranes of solvated, hydrophilic cargo is achieved by evading the hydrophobic milieu, for instance, by shielding the cargo with a membrane during vesicular transport. Clathrin-mediated endocytosis (CME) is probably the best known variant of vesicular import. Besides textbook examples like synaptic vesicles due for recycling [3], nutrients or signaling molecules, intact proteins [4], and intact viruses [5] enter cells by CME. Like in all endocytic transports, a membrane invagination envelopes the cargo. A spherical clathrin superstructure encages the nascent vesicle and attachment to cytoskeletal actin triggers for maturation [6] (see Fig. S1A, Supp. Material for a graphical summary of CME). In spite of some controversy, whether clathrin cages actually induce membrane bending or form on pre-bent membrane areas [[7], [8], [9]], clathrin requires a set of adaptor and regulatory proteins, the exact composition of which controls the eventual size and shape of the vesicles [8,10]. In yeast, adaptor proteins epsin1 and the Hip1R homolog (Sla2) are required to link clathrin to the membrane and to the actin cytoskeleton [11]. Their N-terminal domains, called ENTH (with four isoforms known in yeast, ENTH1 - ENTH4) and ANTH, respectively, recruit lipids and anchor the endocytic machinery to the membrane [12,13] (Fig. S1B, Supp. Material).

Both, ENTH and ANTH, attach to the cell membrane via exposed surface areas of high positive net charge. These interactions have to be strong enough to sustain the considerable straining due to membrane bending [14]. Different supporting mechanisms exist to achieve this. For instance, ENTHs expose an *N*-terminal α-helix, which inserts into the cell membrane (α0 in Fig. S1C, Supp. Material) [12]. Second, the specific binding of phosphatidylinositol-(4,5)-bisphosphate (PIP2) by adaptor proteins triggers their recruitment to the membrane [15]; apparently in concert with the afore-mentioned helix insertion [16,17]. Third, as implied by our recent native mass spectrometry (MS) measurements of ENTH-ANTH mixtures, PIP2 binding is strictly required for oligomerization [13,18] into higher-order assemblies (Fig. S1B, Supp. Material), which would certainly bind stronger to the cell membrane than monomers.

According to these data, PIP2 binding to simultaneously present ENTH1/2 and ANTH monomers induces the formation of 6:6:≈18 complexes, which rapidly convert to 8:8:≈25 complexes (ENTH:ANTH:PIP2, “di-hexamer” and “di-octamer”, respectively) for fungal proteins [18]. Previously, a single PIP2 binding site per ENTH and ANTH-domain was known, while native MS of the single proteins revealed a second PIP2 binding site per protein molecule. Thus, the observed complex stoichiometry falls exactly in between full occupation of one or two binding sites (16 or 32 PIP2 per di-octamer). In combination with the crystal structure of the ENTH2 homodimer containing only one PIP2 ligand [18], which together with the re-opened *N*-terminal α-helix forms the interface for subunit interaction, this implies shared PIP2 binding between subunits within the complexes. ANTH ejection in CID furthermore indicated its peripheral location.

In the human system, ANTH is not necessary at all for inducing higher order oligomers. Instead, binding of six PIP2 ligands proved sufficient to stabilize the ENTH homo-hexamer [18]. Employing the X-ray crystal structure of the yeast ENTH2 homodimer to obtain a structural model for the small angle X-ray scattering (SAXS) data of the human ENTH homo-hexamer yielded a good fit and suggested a ring structure formed by staggered individual subunits (Fig. S1D, Supp. Material). This hexamer proved furthermore to be preferred over the octamer for human ANTH attachment. Taking into account the previously observed cross-species adaptability of subunit interaction [18,19] and assuming the ENTH hexamer to be a preliminary step on the formation of the higher order oligomers, this is perfectly in line with a double ring structure with an inner ENTH and an outer ANTH ring. Each of these rings could then comprise the ‘minimum number’ of eight PIP2 moieties, each shared between pairs of ring members with the ‘surplus’ 8 ligands sandwiched between both rings (Fig. S1B, Supp. Material). This hypothesis, however, warranted further experimental validation, which is the objective of the current manuscript. To achieve this, we applied again native MS, yet extended the array of utilized instruments, fragmentation techniques and included ion mobility (IM). By doing so, we tested our hypothesis regarding complex stoichiometry of the hetero-oligomeric assemblies and the ligand location within them as well as the strength of these interactions and the size of the resulting homo- and hetero-oligomeric complexes.

Proteins can easily impose sample preparation- and data interpretation-related obstacles on structural analysis by conventional methods as X-ray crystallography, EM or NMR due to heterogeneity or lack of stability. Protein structural studies utilizing MS methods tend to circumvent such difficulties because of inherently low sample consumption, high mass accuracy and sensitivity, the theoretical absence of an upper size limit and, above all, the option to measure several simultaneously present association- or conformational states. This is partially true for indirect approaches like surface labelling, backbone amide hydrogen exchange or cross-linking [[20], [21], [22]] and a main feature of direct measurements by native top-down MS. The quantum leap in native MS, however, is the ability to ionize (usually by nano-electrospray) proteins from near-physiological, mostly ammonia-based solutions, which allows for keeping proteins and their complexes in compact native-like conformations with intact crucial non-covalent interactions [23]. Ideally, the subsequent measurement of, and discrimination by, mass provides a snapshot of all simultaneously present non-covalent complexes with masses of up to the order of Mega-Daltons [24] and representative of individual species’ occurrence [25]. Apart from the straightforward quaternary structural status, native MS can provide purely conformational, i.e. tertiary structural data. The key parameter for extracting this information from native mass spectra is charge: The number of protons carried by a protein molecular ion, also after ionization, ultimately correlates to its surface area [[26], [27], [28], [29]] although, as discussed in Ref. [30], there are still ‘heretics’. The yeast ENTH-ANTH di-octamer with its comparatively low precursor ion charge [18] is a case in point. We addressed this and other questions by supplementing data from conventional collision-induced dissociation (CID) [[31], [32], [33], [34], [35], [36]] with surface-induced dissociation (SID). Unlike CID, SID proceeds more quickly, obviates the CID-inherent multiple collisions and thermal processes and, thus, provides structural information independent on individual subunit unfolding [[37], [38], [39]]. Second, we verified our assumptions regarding size and shape of the adaptor protein assemblies by coupling native MS with travelling wave ion mobility (IM-MS) [[40], [41], [42]].

## 2 Materials and methods

**Protein production and purification:** Human ENTH and the yeast ENTH1, ENTH2, and ANTH were produced recombinantly in *E. coli* BL21 DE3 (Novagen) from the pETM30 vector as GST fusion proteins with an *N*-terminal His-tag and a TEV cleavage site between His-GST tag and the protein of interest. Upon cell harvesting, purifications by NiNTA and TEV cleavage followed. For detailed descriptions see Ref. [18]. **Sample preparation for native MS:** Typically, purified proteins were buffer exchanged to 300 mM ammonium acetate (PN 431311, 99.99% purity, Sigma-Aldrich) and 1 mM DL-dithiothreitol (PN 43815, 99.5% purity, Sigma-Aldrich), pH 8.0, via centrifugal filter units (Vivaspin 500, MWCO 5000 and 10000, Sartorius) at 13,000×*g* and 4 °C. Complexes were assembled after buffer exchange by mixing with final concentrations of 10 μM ENTH, 10 μM ANTH and 60 μM PIP2 (Phosphatidylinositol-4,5-bisphosphate diC8, Echelon). **Native Orbitrap MS:** For Orbitrap measurements, yeast proteins were used at a mixing ratio of 0.7 ENTH1: 1 ANTH: 3.5 PIP2 and exchanged into ammonium acetate after complex assembly [13]. Samples were analyzed by nano-electrospray ionization using gold-coated borosilicate capillaries prepared in-house. An aliquot of 1–2 μl was loaded into the capillary and sprayed in positive ion mode at a potential of 1.25 kV in the source of an Exactive Plus Orbitrap customized prototype modified for native MS as described previously [43,44]. The instrument was operated with xenon as collision gas in the HCD cell, with the gas pressure optimized for ion transmission to a read back of 0.5-1 × 10^−9^ mbar in the ultra high vacuum region of the Orbitrap mass analyzer. Source fragmentation parameter and collision energy in the HCD cell, both to force desolvation of the protein complex ions, were 100 V and 50 V, respectively. Transients were recorded for 64 ms per scan, averaging over several minutes to acquire the spectra presented. **Native QToF1 MS:** Yeast proteins were used at a mixing ratio of 0.7 ENTH1: 1 ANTH: 3.5 PIP2 (16 μM, 23 μM, and 81 μM) and buffer exchanged into ammonium acetate, either before or after complex assembly [13]. Samples were analyzed by nano-electrospray ionization using gold-coated borosilicate capillaries prepared in-house. An aliquot of 1–2 μl was loaded into the capillary and sprayed in positive ion mode at 10 mbar source pressure. 1.5 × 10^−2^ mbar xenon was used as collision gas. Collision voltage was 75 V. Capillary and sample cone were at 1350 V and 150 V, respectively. **Native ion mobility MS:** Proteins were prepared as described and introduced into a commercially available travelling wave ion mobility mass spectrometer (Synapt G2™ HDMS, Waters) by nano-electrospray using home-made gold-coated borosilicate emitters. The instrument is equipped with a 32 k quadrupole allowing transmission of high mass ions and with a pusher frequency of 1900 s^−1^. All measurements were done in positive ion mode. The highest values used for measuring yeast ENTH:ANTH:PIP2 complexes and still considered native were 2.3 kV, 50 V, and 0.5 V capillary, sample cone, and extraction cone voltage, respectively. Trap and transfer collision energies were 15 V and 5 V, trap DC bias and helium DC bias 45 V and 25 V, respectively, if not stated otherwise. Backing pressure was set to 8 mbar and 2.5 mbar nitrogen (60 ml/min IMS gas flow) was used for ion mobility separation. For ion separation, wave velocities of 650 m/s and wave heights of 40 V were used. Wave velocities in trap and transfer cell were 300 m/s and 64 m/s with wave heights of 6.0 V and 2.9 V, respectively. Collision gas was argon with 4.6 × 10^−2^ mbar and 4.4 × 10^−2^ mbar in trap and transfer cell, respectively. Settings for measurements of human PIP2-containing ENTH hexamers were 1.3 kV capillary voltage, 25 V sampling cone, 4 V extraction cone voltage. Trap collision energy, trap DC bias, helium DC and transfer collision energy were set to 25 V, 45 V, 25 V, and 5 V, respectively. Backing pressure was 8.5 mbar and 3.1 mbar nitrogen (90 ml/min IMS gas flow) was used for ion mobility separation. Argon was used as collision gas with 3.1 × 10^−2^ mbar in the trap and 3.2 × 10^−2^ mbar in the transfer cell. Travelling waves with heights of 25 V and velocities of 300 m/s were used for ion mobility separation. Wave velocities in trap and transfer cell were 300 m/s and 66 m/s with wave heights of 6.0 V and 2.0 V, respectively. Alcohol dehydrogenase from *S. cerevisiae* (#A7011), concanavalin A from *C. ensiformis* (#C2010), glutamate dehydrogenase from bovine liver (#G7882), pyruvate kinase from rabbit muscle (#P9136), and bovine serum albumin (#P7656), all from Sigma-Aldrich, were used as ion mobility calibrants employing a logarithmic fit procedure [45,46] and measured under identical conditions as the proteins of interest. The theoretical CCS of human ENTH hexamer SAXS model was calculated using IMos [47] with the projection approximation (PA) algorithm at 298 K and He as a buffer gas. To correct for the use of N_2_ and the according underestimation, CCS was scaled by the experimentally derived factor of 1.14 [48]. **Surface-induced dissociation MS:** Samples were prepared and sprayed as described above and analyzed in positive ion mode on a Synapt G2™ HDMS instrument with an additional SID cell located upstream of the ion mobility separation cell [39]. The SID cell contained a gold surface coated with 2-(perfluorodecyl)ethanethiol [49]. Spectra in transmission mode, *i.e.* without a collision with the surface, were acquired with 1.6 kV capillary voltage and 80 V sampling cone voltage. Trap and transfer cell collision energies were both set to 10 V. 2.5 mbar nitrogen was used for ion mobility separation. Travelling waves with heights of 20 V and velocities of 300 m/s were used in the IMS cell. Measurements in SID mode were performed with 1.8 kV capillary voltage and 50 V sampling cone voltage. Trap and transfer collision energies were set to 10 V and 4 V, respectively. 2.2 mbar nitrogen was used for ion mobility separation of SID products in the IMS cell. The potential difference between the trap DC bias and the surface of the SID cell defined the SID collision energy. It was ramped from 30 V upwards. Due to the low signal intensity in MS/MS mode, spectra with broader precursor selection windows were acquired. For that purpose, the MS profile was set to the range of 10,000–15,000 *m/z* for 75% of the dwell time.

## 3 Results

### 3.1 Direct characterization of the yeast ENTH1-ANTH-PIP2 complexes by native MS

As outlined above, PIP2 binding to individual fungal ENTH or ANTH does not change the monomeric state of either protein. However, as soon as both proteins and PIP2 are present, ligand binding induces formation of a rather low-abundant di-hexamer, which quickly grows into the prominent di-octamer. Formation of these complexes proceeds in a highly cooperative manner as no other intermediate species accumulated [18] (see also Figs. S2 and S3, Supp. Material). Furthermore, complex stoichiometry is unaltered if protein ratios are distinct from 1:1 or less PIP2 is present in solution (Fig. S3, Supp. Material). Reduction in PIP2 amount however reduced the complex abundance. A further intriguing aspect of these data were the rather low charge states centering at 33/34 + and 37/38 + for di-hexa- and di-octamers, respectively. Estimates of the charge states from an empirical correlation ($z=0.0467\times \sqrt[{1.8868}]{\text{MW}}$) [50] predict substantially higher charge states of 39 + and 45+, respectively. Intriguingly, we had observed somewhat different values (41/42+) before [13] (see also Fig. S3, Supp. Material). These differences are small but important, since the latter data had been recorded in an instrument with a somewhat more gentle ion source (Q-ToF1) than the former (Q-ToF2) but revealed a higher charge nevertheless. Importantly, an opposed trend of carrying less PIP2 ligands with increasing charge is observed (Q-Tof2: ≈26 PIP2, Q-ToF1:≈24 PIP2), which in turn, is independent of the actual concentration of PIP2. A reasonable question is therefore, if some of the PIP2 ligands (acidic in solution) had shielded potentially charged residues and, if so, where this had been occurring.

**Fig. 1:**
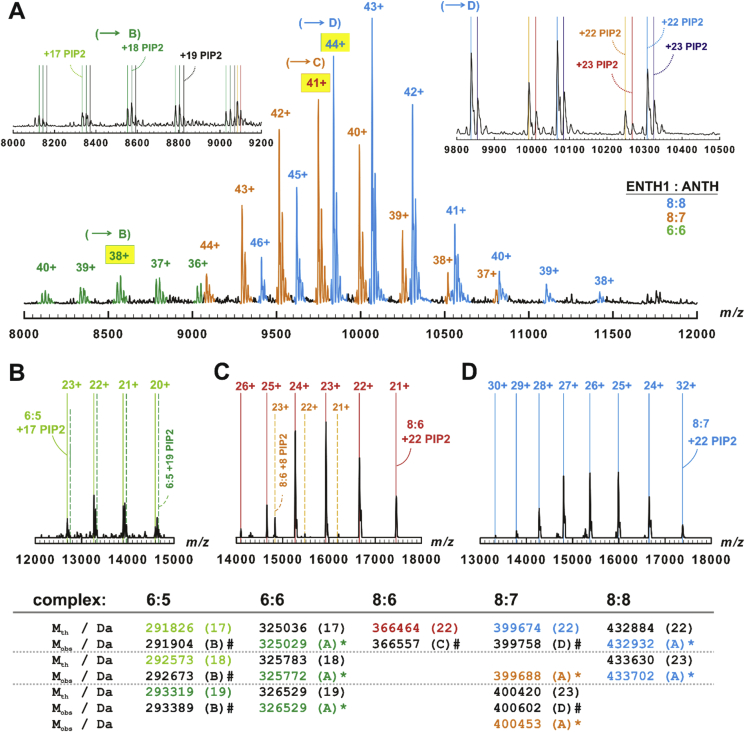
**Higher order complex formation of yeast ENTH1 and ANTH. A:** Orbitrap mass spectrum of yeast ENTH1 and ANTH assembling into di-hexamers, pentadecamers (8:7) and di-octamers (green, orange and cyan peaks, respectively). Capital letters and hatched-in charge labels denote the peaks selected for MS/MS (**B-D**). *Insets:* Lower (left) and upper (right) parts of the spectrum with theoretical *m/z* of the di-hexamer (green) as well as of the pentadecamer (8:7) (orange, red) and the di-octamer (cyan, blue) with 17–19, 22 or 23 PIP2 ligands indicated by vertical lines. **B-D**: Relevant parts of fragment ion mass spectra of the di-hexamer (**B**), the pentadecamer (8:7) (**C**), and of the di-octamer (**D**) complexes. Vertical lines denote the theoretical *m/z* of hexa-pentameric (**B**), of octa-hexameric (**C**), and pentadecameric (8:7) stripped (**D**) complexes with the indicated number of PIP2 ligands. Lower panel: comparison of theoretical (*M*_*th*_) and experimentally determined (*M*_*obs*_) masses. The font color mirrors measured (**A**) or theoretical (**B-D**) *m/z*; numbers in brackets indicate the amount of PIP2 ligands. #: determined by MS/MS. *: determined from two independent precursor ion mass spectra, difference was always below 0.5 Da.

Exploiting the better desolvation and therefore higher resolution of an Orbitrap instrument to directly derive the number of lipids confirmed our previous findings with some small but important differences observed for the di-hexamer and di-octamer compared to QToF data (compare Fig. 1A, Fig. S2, S3 and S4, Supp. Material). First, the number of observed ligands decreased slightly to 17–18 for the di-hexamer and to 22–23 for the di-octamer (Fig. 1A). Second, the predominant charges increased to 37/38 + and 43/44 + for di-hexa- and di-octamer respectively. These values are much closer to the predicted ones but also to those measured in the Q-ToF1. This, together with the similarly decreased number of ligands, tends to corroborate the assumption of PIP2 ligands shielding potentially charged residues. Third, we could detect and clearly identify a further, intermediate species consisting of eight ENTH1 and seven ANTH subunits carrying the same amount of 22–23 PIP2 ligands as the di-octamer (pentadecamer (8:7)). Because of the charge state, this cannot be a CID product. Similar species were occasionally observed at lower PIP2 contents (unpublished data CU) and most likely result from imperfect complex assembly or lifetime extension of an intermediate state.

Next, we selected peaks representing one of the complex stoichiometries for dissociation by collisional activation. As is typical for CID in native MS, this resulted in the most easily unfolded and detached subunit being expelled, taking away a disproportionally high fraction of charges (Fig. 1B-D). Importantly and in line with our previous data [18], the detached component was an ANTH subunit in all three cases, whilst all PIP2 ligands remained with the stripped complex. The sole exception being the intermediate pentadecamer (8:7) complex: Here we could observe a minor populated product, which we tentatively identified as a tetradecamer (8:6) stripped complex with eight PIP2 ligands. The main dissociation product is a tetradecamer (8:6) with 22 lipids, which was already observed in the overview spectra (Fig. S4A inset, Suppl. Material). Under these conditions neither the di-octamer nor di-hexamer dissociated indicating reduced stability of the 8:7 intermediate. Due to the lower stability, we considered alternatively tetradecamer (8:6) complexes after ANTH loss with additional backbone cleavages, *i.e.* with single covalent fragments of either ENTH1 or ANTH lost and *vice versa* more PIP2 ligands retained. DY breaks in ENTH1 plus 1–2 PIP2 losses were close to the observed mass and have a high propensity according to Ref. [51]. Hence, these species and the unusual 8:6:8 complex are the most likely assignments. The actual instrument settings did not allow detection of the detached high-charged species or lipids (*i.e.* low *m/z* products, see Fig. S4, Supp. Material) but a single ANTH is unlikely carrying away 14 ligands. Nevertheless, this would indicate an opening up of the interface is necessary to release lipids from the complex and eight retained ligands equal the number of ENTH1 subunits in this complex. ENTH1 as inner ring subunit is always protected in full assemblies in CID, hence only an 8:7 precursor would allow early unfolding of ENTH1 and hence backbone fragmentation. Both assignments would confirm our original hypothesis of an inner, PIP2-stabilized ENTH core.

### 3.2 Characterization of the yeast adaptor complexes by IM-MS

As a means to gain information about the architecture of the higher order assemblies, we also measured ion mobilities using a Synapt G2 HDMS™ instrument. By doing so, we found a QToF2-like pattern with respect to charge states and ligand binding (compare Figs. S2C and S5A, Supp. Material): the most intense peaks representing di-hexamers with 21 or 22 and di-octamers with 26 PIP2 ligands assuming all additional mass to be lipids centered at 34 + to 35 + and 38+. In addition, we observed and tentatively assigned an intermediate species consisting of six ENTH2 and three ANTH subunits that carried 18–20 PIP2 ligands (nonamer (6:3), Fig. S5, Supp. Material) likely due to an incomplete assembly as the charge state distribution does not support gas phase dissociation. Despite the reduced number of ANTH subunits, the amount of ligands is still comparatively high (≈2 per protein subunit), which tends to confirm again our original hypothesis of an inner ENTH and an outer ANTH ring with some of the ligands localized between both rings.

**Fig. 2:**
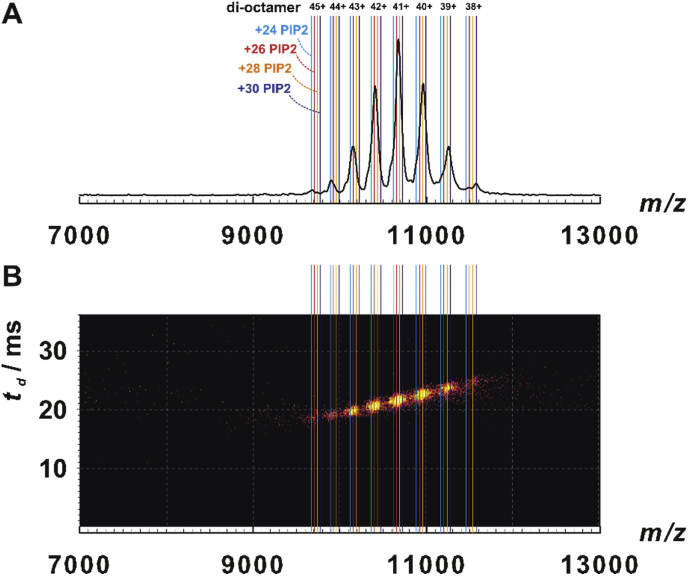
**Higher order complexes of yeast ENTH1 and ANTH. A:** Mass spectrum of yeast ENTH1-ANTH-PIP2 di-octamers detected after applying MS profile (favoring 10,000–15,000 *m*/*z*). **B:** Heat map combining MS (horizontal) and ion mobility (vertical) dimensions. The intensity scale is logarithmic. As in **A**, vertical lines denote theoretical *m*/*z* of di-octamers carrying the indicated number of PIP2 ligands.

However, the intensity of both major complexes was low with the di-hexameric species being more prominent. This, and the observation of the intermediate nonameric (6:3) species alike, may result from the Synapt having decreasing transmission at higher *m/z.* Another reason could be sample quality or suboptimal electrospray, nevertheless, the overall sample behavior was unaltered. Whatever the reason, the low intensity hampered MS/MS experiments of di-octamer complexes. The application of an MS profile (enhancing 10,000–15,000 *m/z*) for ENTH1-ANTH complexes favored transmission of di-octamers carrying a comparable number of ligands as before (Fig. 2A) and was therefore employed in dissociation studies of the large complexes (section 3.3). The signals of most charge states in the 2D IM-MS plots appeared compact and well-defined (Fig. S5B, Fig. S6, Supp. Material and Fig. 2B). The collision cross sections (^TW^CCS_N2_) for all relevant ENTH-ANTH complexes could thus be calculated (Fig. S7, Supp. Material, Table 1).

**Table 1:** **Collision cross sections of yeast ENTH-ANTH complexes.** *: measurements with ENTH1 and with MS profile applied. Roman numbers refer to the ensembles referred to in Fig. S7, Supporting information.

complex	*z*	t_d_/ms	^TW^CCS_N2_/Å^2^
6:3 (I)	30	13.12	10,260
	29	13.85	10,110
	28	14.94	10,040
6:6 (II)	36	16.58	13,407
	35	17.50	13,295
	34	18.41	13,157
	33	19.50	13,042
	32	20.78	12,944
8:8 (III)	39	22.05	16,121
	38	23.15	15,989
	37	24.42	15,876
	36	25.88	15,778
8:8 (IV)*	44	18.95	17,210
	43	19.68	17,050
	42	20.41	16,880
	41	21.69	16,850
	40	22.60	16,680
	39	23.69	16,550
	38	24.97	16,440

Without solution or crystal structures for calculating theoretical CCS values, ^TW^CCS_N2_ values of reference proteins [45] can be used to predict the compactness of our complexes [50]. Some of these proteins were used for calibration, thus introducing some bias in this comparison with respect to the mass–CCS–relation. Nevertheless, it revealed some findings of interest. Within the reference dataset of native proteins and protein complexes, only the smallest protein showed some dependence of ^TW^CCS_N2_ on charge - the larger the ions, the lesser the influence of actual charge on their ^TW^CCS_N2_. The ^TW^CCS_N2_ of the yeast ENTH-ANTH complexes, on the other hand increased slightly but significantly with charge (Fig. S7, Supp. Material). This becomes most obvious by comparing the data of the ENTH2-ANTH di-hexamer with those of the glutamate dehydrogenase (GDH) hexamer. With masses of 319 kDa (ENTH2-ANTH) and 336 kDa (GDH) as well as ≈13,200 Å^2^ and ≈13,400 Å^2^, respectively, both complexes are highly similar, yet their predominant charge states differ with 33+/34 + for ENTH2-ANTH di-hexamer complexes and 36 + for GDH [45]. Note that the charge state of the di-hexamer from Orbitrap measurements does match the charge state of GDH. We conclude that the ENTH2-ANTH di-hexamer complex in the gas phase is compact but not more compactly folded than expected for globular proteins and rather gains exceptionally few charges for its surface area in the actual ion source. This also holds for the other ENTH:ANTH complexes.

### 3.3 Characterization of the human ENTH hexamer by IM-MS

As in our previous measurements [18], human ENTH oligomerized also in the absence of ANTH, PIP2 alone being required for complex formation. In addition to ENTH hexamers, peaks representing a complex mixture of monomeric and dimeric proteins with up to two bound PIP2 ligands were highly abundant on the Synapt (Fig. S8 Supp. Material, labeled “I” and “III”). We observed this behavior previously on QToFs but to a lesser extent and independently of the exact measurement conditions; so it again may result from suboptimal transmission. Peak intensities of the complexes nevertheless proved sufficient for IM-MS and ^TW^CCS_N2_ calculation (Figs. S8 and S9, Supp. Material). Table 2 shows the values obtained from spectra measured at mild conditions (25 V in trap cell). Ranging from ≈6480–6650 Å^2^, they are reasonably similar to the 6270 Å^2^ that we expected from our SAXS model (Fig. S1D Supp. Material) [18]. The difference of approximately 300 Å^2^ most probably results from the fact that the unresolved C-terminal amino acids of the subunits were modeled as dummy-glycines. Therefore, the complex is in fact slightly larger than implied by the SAXS model. In addition, we observed a distinct behavior compared to yeast ENTH-ANTH complexes for the ensemble itself. The discrete ^TW^CCS_N2_ values increased significantly less with charge state. Moreover, the ^TW^CCS_N2_ values and charge states of the ENTH hexamer were similar to the ^TW^CCS_N2_ of the calibrant corresponding in mass, concanavalin A (Fig. S7, Supp. Material).

**Table 2:** **Collision cross sections of human ENTH-hexamers.** Roman number refers to the ensembles referred to in Fig. S7, Supp. Material.^a^: second, more compact conformation (see main text).

complex	*z*	t_d_/ms	^TW^CCS_N2_/Å^2^
6:0 (V)*	23	11.66	6650
	22	12.39	6570
	21	13.30	6520
	20	14.40	6480
	19	15.86	6480
	20	12.94^a^	6,120^a^
	19	14.22^a^	6,110^a^

**Fig. 3:**
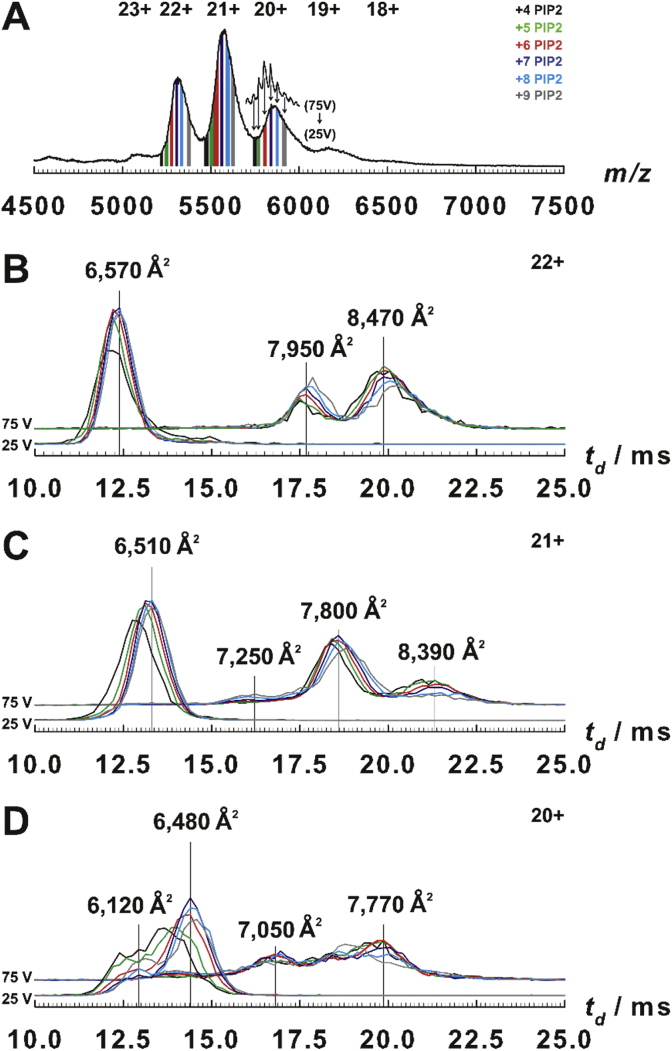
**PIP2-dependent conformations of human ENTH hexamer. A:** Mass spectra (acquired with 25 V in the trap cell of the Synapt) comprising the hexamer signals. Hatched-in areas represent the complex with PIP2 load as indicated and denote the *m*/*z* ranges used for ATD extraction as derived from the 75 V spectrum. To illustrate *m*/*z* range used the 20 + peak of the 75 V spectrum is depicted as inset with arrows denoting the ligand binding states as used for extraction of ATDs from the 25 V spectrum. **B-D:** ATDs representing the 22+, 21 + and 20 + charge states carrying 4–9 (see **A** for colors) PIP2 ligands. ATDs were normalized to total intensity, with those acquired with 75 V, offset by 2% along the y-axis. Vertical lines specify the drift times corresponding to the above-written CCS’s. (For interpretation of the references to color in this figure legend, the reader is referred to the Web version of this article.)

The actual peak shape was entirely devoid of fine structure (Fig. 3, Fig. S9A in Supp. Material) until collisionally activated (75 V in trap cell). Nicely resolved peaks represented hexamers with 4–9 PIP2 ligands each (Fig. S9B in Supp. Material). Under these activating conditions, the ATDs reveal that the complex clearly underwent substantial unfolding, without, however, subunit dissociation (compare Figs. S9A and B, Supp. Material). So the resolved peaks were used to define the *m/z* range for a given number of PIP2 bound and extract ligand binding-specific ATDs from the native 25 V spectrum (Fig. 3A). Intriguingly, the drift times shifted slightly upwards with each subsequently bound PIP2 from four until six to seven ligands by about 40–50 Å^2^ per added PIP2 (Fig. 3B-D). As complexes devoid of PIP2 cannot be produced experimentally, we estimated the theoretical ^TW^CCS_N2_ with 6144 Å^2^ from the model structure after PIP2 deletion assuming no conformational changes. An increase of 21 Å^2^ per PIP2 ligand would be expected purely due to the additional volume of the ligand. The actually observed somewhat larger ^TW^CCS_N2_ increase probably does not result from any unfolding event for two reasons: First, unfolding of the hexamers proceeds in larger steps of at least 400 Å^2^, as shown by the collisionally activated species. Second, while shifting, the peaks symmetrically sharpen and gain intensity (Fig. 3B, C) indicating a more regularly folded structure. We conclude therefore, that only an approximately stoichiometric ligand to subunit ratio ensures proper folding. Further evidence supporting this conclusion comes from the ATDs of collisionally activated ENTH hexamer. A closer look reveals that unfolding is increasingly impaired with more PIP2 ligands bound (Fig. 3B-D). This, in turn, hints at a stabilization of folded complexes by ligand binding. Additionally, we observed an interesting bimodal distribution of the low-charged (19 + and 20+) folded entities (Table 2 and Fig. 3D). Although theoretically possible, it is unlikely that this peak splitting results from a local or global unfolding in the strictest sense. More probably, parts of the complexes have undergone a slight collapse during or upon ionization [48]. Such a compaction of ≈360 Å^2^ would nicely agree with an internal cavity as seen in the SAXS model (Fig. S1D, Supp. Material). In line with the ^TW^CCS_N2_ increase seen with the higher charged species, this compaction furthermore appears impaired with ligand binding.

### 3.4 SID-measurements of yeast ENTH1-ANTH di-octamer complexes

**Fig. 4:**
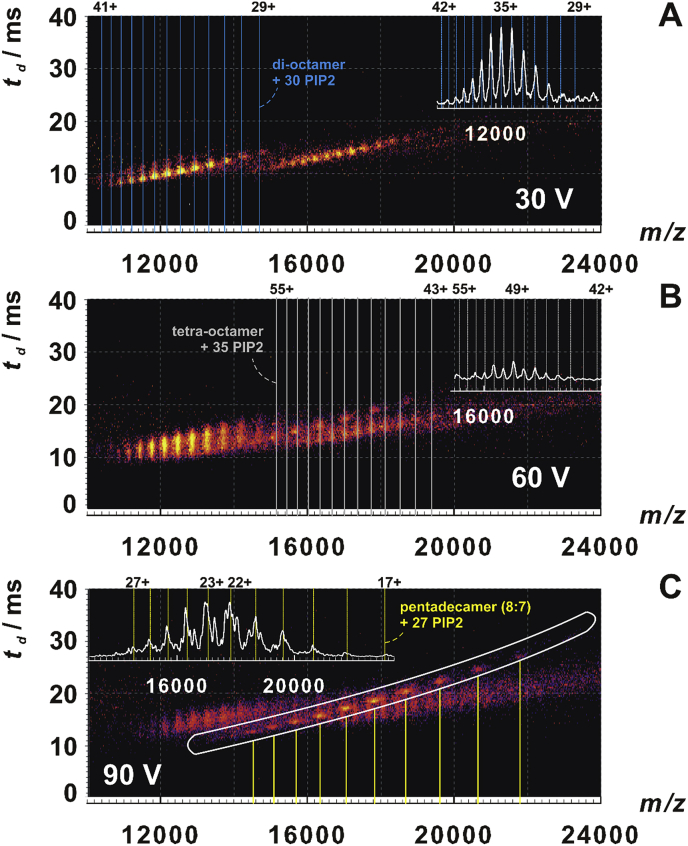
**Surface induced dissociation (SID) of yeast ENTH1-ANTH di-octamers.** SID mass spectra acquired with MS profile favoring 10,000–15,000 *m*/*z* and with the indicated SID voltages shown as heat maps combining MS (horizontal) and ion mobility (vertical) dimensions. The intensity scale is logarithmic. Vertical lines denote theoretical *m*/*z* of di-octamers, (**A**), of tetra-octamers with 35 PIP2 ligands (**B**), as well as of pentadecamers (8:7) (**C**), each with the indicated number of PIP2 ligands. Insets show MS traces of the corresponding parts of the spectra or of the excised and separately evaluated region encircled in white (**C**).

In order to dissect complex topology further, we employed SID, which alters dissociation pathways compared to CID. Peak intensities of yeast di-octamer ENTH1-ANTH complexes were too low to perform SID in combination with precursor ion selection. Therefore, we measured in MS mode with SID voltages increasing from 30 V to 120 V. Using this approach under mild conditions (30 V in SID), we observed as dominant species the di-octamer complex with 22–31 ligands and a previously described aggregate [18], a dimer of the di-octamer or ‘tetra-octamer’ (Fig. 4, Fig. S10 Supp. Material). There is, however, a visible ATD tailing of the di-octamer peaks (Fig. 4A, Figs. S10 and S12 Supp. Material) hinting at conformational expansion under mild conditions. At increased SID voltages, this develops into a substantial peak shift, which is, however, distinct from complete unfolding (60 V, Fig. 4B, Figs. S11 and S12 Supp. Material). Under these conditions, free ANTH products appeared while the di-octamer peak intensities decreased. In the tetra-octamer region an additional species appears (Fig. 4B). The 2D heat maps (Fig. 4C, Fig. S11 Supp. Material) allowed extraction and identification of this newly emerged species as pentadecameric (8:7) dissociation product with 27 attached ligands (inset in Fig. 4C). Although produced without quadrupole selection, both, the pentadecamer (8:7) stripped complex and the released ANTH were clearly identifiable as dissociation products of the di-octamer amongst others by their increasing appearance with ramped-up SID voltage (Fig. 4A-C, Figs. S10 and S11 Supp. Material). Further evidence comes from their complementary charge state patterns: The highest observed charge states were 27 + and 17 + for pentadecamer (8:7) and ANTH, respectively; the most intense ones were 23 + and 11+, both of which added-up nicely to the highest (42+) and the most intense (34+) charge states, respectively, of the di-octamer precursors. As opposed to CID, released ANTH subunits occasionally carried a single PIP2 along (Fig. 4B), which indicates partially retained fold and direct interaction between ANTH and PIP2 in the complex.

## 4 Discussion

The preferred charge and ligand-binding states of the yeast ENTH-ANTH di-octamers are a special case: As outlined, theory predicts 45 + as dominant species. Although, membrane proteins may be expected to charge up less in presence of lipids, at least when well resolved they usually match the prediction. The membrane-associated complexes described here almost reached this value (43+) on an Orbitrap instrument as opposed to QToF or Synapt measurements. It is tempting to deduce these differences to peculiarities of the concerned instruments or spray conditions; however, charge states inversely correlate with lower ligand binding; the extremes being 23 PIP2 at 43+ (Orbitrap) and up to 31 PIP2 and 34+ (Synapt, SID-IM-MS). This also holds on spectrum level. In Fig. 1, the ratio of the peaks representing either 22 or 23 ligands increases in favor of the latter with decreasing charge. Moreover, the peak apexes shift in lower resolution data suggesting higher PIP2 load for lower charge states. Therefore, PIP2 binding reduces the net charge of the ENTH-ANTH complexes.

The simplest explanation for this finding is neutralization of positively charged residues by the negatively charged phospholipid. Charged residues include arginine and lysine residues, which are abundant close to the previously known canonical ligand-binding sites. Neither for the monomers nor the human ENTH hexamer, however, was the PIP2 induced charge reduction observed suggesting that it is specific for the higher order oligomers. This in turn implies that the PIP2-induced charge reduction is due to surplus ligands residing at ANTH-ENTH interfaces. Thus, surficial arginine and lysine residues between outer and inner ring subunits are good candidates. Although of highly speculative nature, this may prompt future modelling attempts. Such surficial lipids cannot be bound as tightly as the CID resistant ones and could be regarded as gradually increasing membranous environment. Upon release of membrane proteins from membrane environments, lipids are gradually stripped off [52] (see also Fig. S5, in Ref. [53]). With increased lipids in specific positions as shown here, this suggests that not only structural but also annular belt lipids can be studied by native MS. The results provide further evidence that even though charge is a good estimate of surface area and retention of native-like structure, membrane-associated complexes like transmembrane complexes can exhibit lower charge [54,55].

The human ENTH hexamer or inner ring also exhibits a compact structure with ^TW^CCS_N2_ values in agreement with the SAXS model. Importantly, an observed slight compaction of the hexamer would fit to a cavity as inferred from the SAXS model (Fig. S1D, Supp. Material). Apparently, the native-like conformation is becoming more rigid and stabilized by PIP2 binding indicated by minor ^TW^CCS_N2_ increase and sharpening of the ATDs. Notably, the yeast complexes, all of which contained ANTH subunits in addition to the ENTH1/2 core, while still compact, appeared to be slightly less rigid (Fig. S7, Supp. Material). This suits the finding of further complexes besides the previously described di-hexamer and di-octamer in several experiments for the yeast ENTH-ANTH. In all cases, the complexes lacked ANTH subunits (6:3, 8:7), whereas the ENTH stoichiometry was strictly limited to hexameric and octameric. This is further support for a metastable ENTH core also in yeast, onto which the ANTH subunits assemble. The 6:3 and 8:7 complexes further exemplify that indeed lipids reside at the double ring interface, as these assemblies are essentially fully lipid loaded. The pentadecamer (8:7) moreover nicely fits to the transition from di-hexamer to di-octamer [18]. The observed reduced stability suggests that an even number of yeast ENTH and ANTH subunits is needed to yield a robust assembly. Importantly, major differences in stoichiometry including PIP2 load were not observed even at altered assembly ratios or fast PIP2 depletion from solution, hinting at significance of the complexes and strong cooperativity in the complexes.

SID was employed to gain further insight into the complex structure. Originally, we hoped for half rings and such being observed as described for similar architectures [56] but only individual ANTH subunits were ejected, which retained little or no PIP2. As SID is quicker and not relying on vibrational heating, ANTH subunits should not unfold as strongly as in CID and hence retain their ligands. SID-released ANTH (most intensive peak: 11+, Fig. S11C, Supp. Material) indeed resembles natively sprayed free or singly PIP2-bound protein (most intensive peak: 10+, Fig. S2B, Supp. Material) much more than CID released ANTH (most intensive peak: 18+ [18]). To conclude, weak or entirely absent interactions between the surficial ANTH subunits (as opposed to tightly packed ENTH core) could have resulted in the observed release of single ANTH monomers.

While the stripped complex carried fewer PIP2 (≈27) than the precursor (≈31), only a small fraction of the released SID-ANTH subunits had a bound lipid (Fig. S11C Supp. Material). The released PIP2 itself was not observed. A deflected flight path due to the actual SID settings, further fragmentation, neutral or negatively charged loss could be potential explanations. The remaining question is the PIP2 dissociation itself. As discussed above, the loss of surficial lipids located at the double ring interface does not require subunits to unfold. Assuming that the individual subunits forming both rings remain folded, the observed ^TW^CCS_N2_ increase prior to and during actual complex dissociation conceivably results from an opening of the still intact rings. Such loosening of the quaternary structure can easily lead to the described loss of the ‘surplus’ or ‘inter-ring’ ligands. Moreover, the required minimum number of 22–23 PIP2 as observed in Orbitrap CID is still present in the remaining complex. Such lipids are shared between multiple subunits and hence would be tightly bound. As such, the probability of a released ANTH to carry along a PIP2 is low. Albeit unusual for SID, the dissociation behavior is therefore fully in line with our initial hypothesis and with interfacial lipids mediating the ENTH-ANTH interaction.

To conclude, our results further confirm the proposed assembly model with a ring-shaped ENTH core surrounding a central cavity. PIP2 lipids mostly locate to the interface of the ENTH and ANTH subunits, which is in line with little PIP2 carried along by SID-ejected ANTH subunits, PIP2-induced charge reduction exclusively in ENTH-ANTH complexes and high lipid content in complexes missing ANTH subunits. The additional complex stoichiometries are supportive of the fungal complexes also assembling around an ENTH core, which is however not stable at room temperature in absence of ANTH [18]. SID furthermore suggests ANTH subunits barely interact with each other, while the 8:7 intermediate points to direct ANTH interaction or ANTH requirement for ENTH core stabilization.
