# Supplementary material for: Further insights from structural mass spectrometry into endocytosis adaptor protein assemblies
Source: Int J Mass Spectrom. 2020 Jan;447:116240. doi: 10.1016/j.ijms.2019.116240 (PMC7116418; doi:10.1016/j.ijms.2019.116240)
Supplement: Multimedia component 1 [file mmc1.pdf]

Further insights from structural mass spectrometry into endocytosis adaptor protein assemblies

Johannes Heidemann[1], Knut Kölbel[1], Albert Konijnenberg[2], JeroenVan Dyck[2], Maria Garcia-Alai[3], Rob Meijers[3], Frank Sobott[2, 4], Charlotte Uetrecht [1, 5]

Affiliations: [1]:Heinrich Pette Institute Leibniz Institute for Experimental Virology, Martinistrasse 52, 20251 Hamburg, Germany.
[2]: University of Antwerp, Biomolecular & Analytical Mass Spectrometry, Chemistry Dept. Campus Groenenborger V4, Groenenborgerlaan 171 2020 Antwerp, Belgium.
[3]: European Molecular Biology Laboratory (EMBL), Hamburg Outstation, Notkestrasse 85, 22607 Hamburg, Germany.
[4]: Astbury Centre for Structural Molecular and Cellular Biology, University of Leeds, Leeds LS2 9JT, United Kingdom&School of Molecular and Cellular Biology, University of Leeds, LS2 9JT, United Kingdom.
[5]: European XFEL GmbH, Holzkoppel 4, 22869 Schenefeld, Germany
Correspondence and requests for materials to C.U. (email: charlotte.uetrecht@xfel.eu)

Supplementary Material:

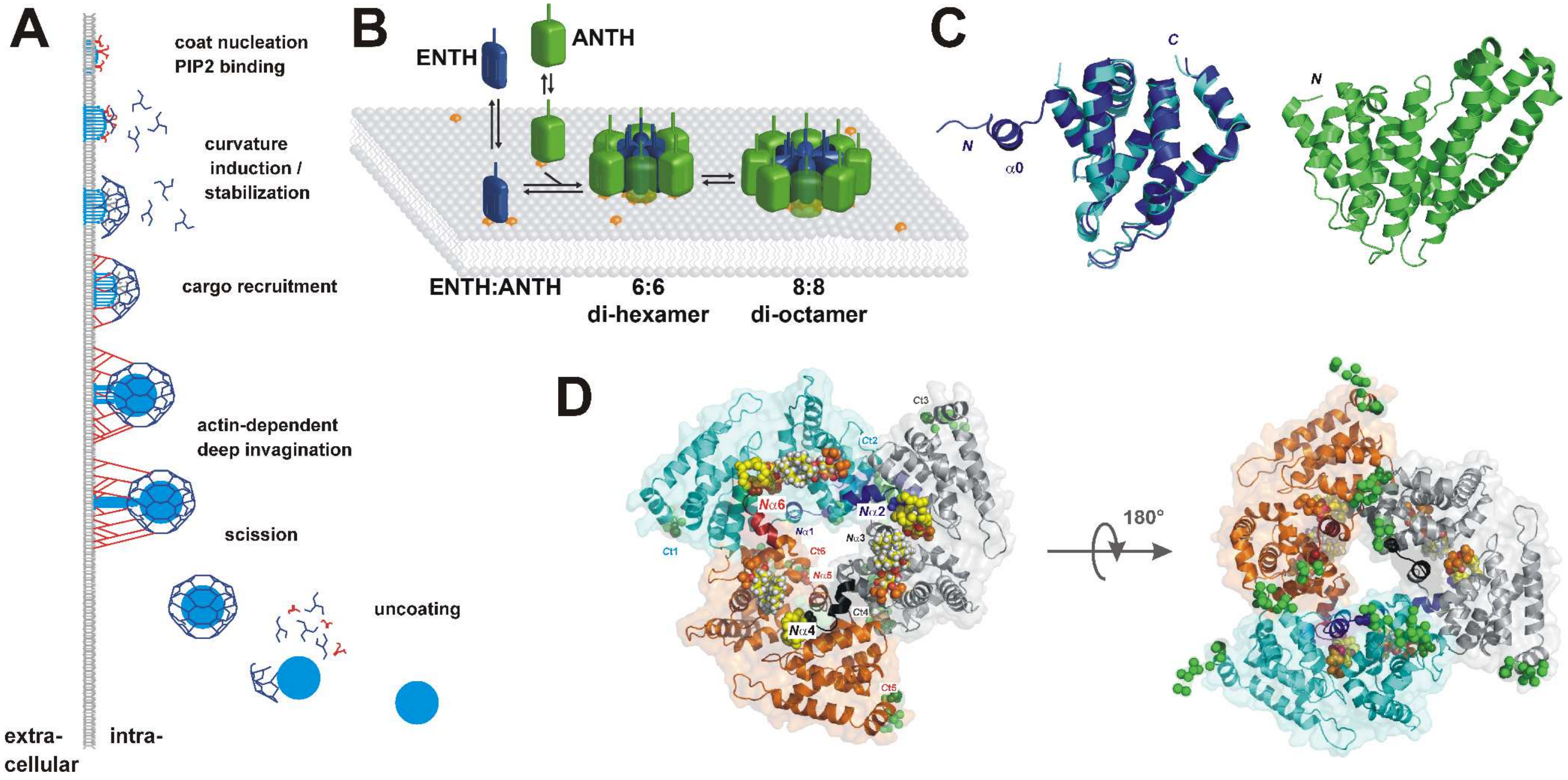

Figure S1Clathrin-mediated endocytosis and adaptor proteins.A: Schematic view of endocytic progress. Adapted from [1]. **B:**Schematic view of yeast adaptor proteins and their assemblies. Adapted from [2]. **C:** Aligned tertiary structures of yeast ENTH1 (PMID 5onf, blue) and ENTH2 (PMID cyan, yellow) as well as of *Chaetomium thermophilum* ANTH (homologous to the *S.cerevisiae* protein, PMID 5oo7, green). **D:** SAXS model of human ENTH homo-hexamer [2] with two protein subunits colored cyan, orange and grey, each and their *N*-terminal ten amino acids emphasized in blue, red and black, respectively. Green spheres represent *C*-terminal dummy residues; yellow ones the C-atoms of bound dioctanoyl l-α-phosphatidyl-d-myo-inositol 4,5-diphosphate (PIP2). To facilitate discrimination of the six different PIP2, three of them are shown with hydrogens present, the other three with hydrogens deliberately removed. Labels specify *C*-termini (*Ct*) and *N*-terminal α-helices (*N*α) of individual subunits indicated by Arabian numbers. Larger font size and respectively darker colors of the backbone indicate *N*-terminal α-helices pointing out of the paper plane.

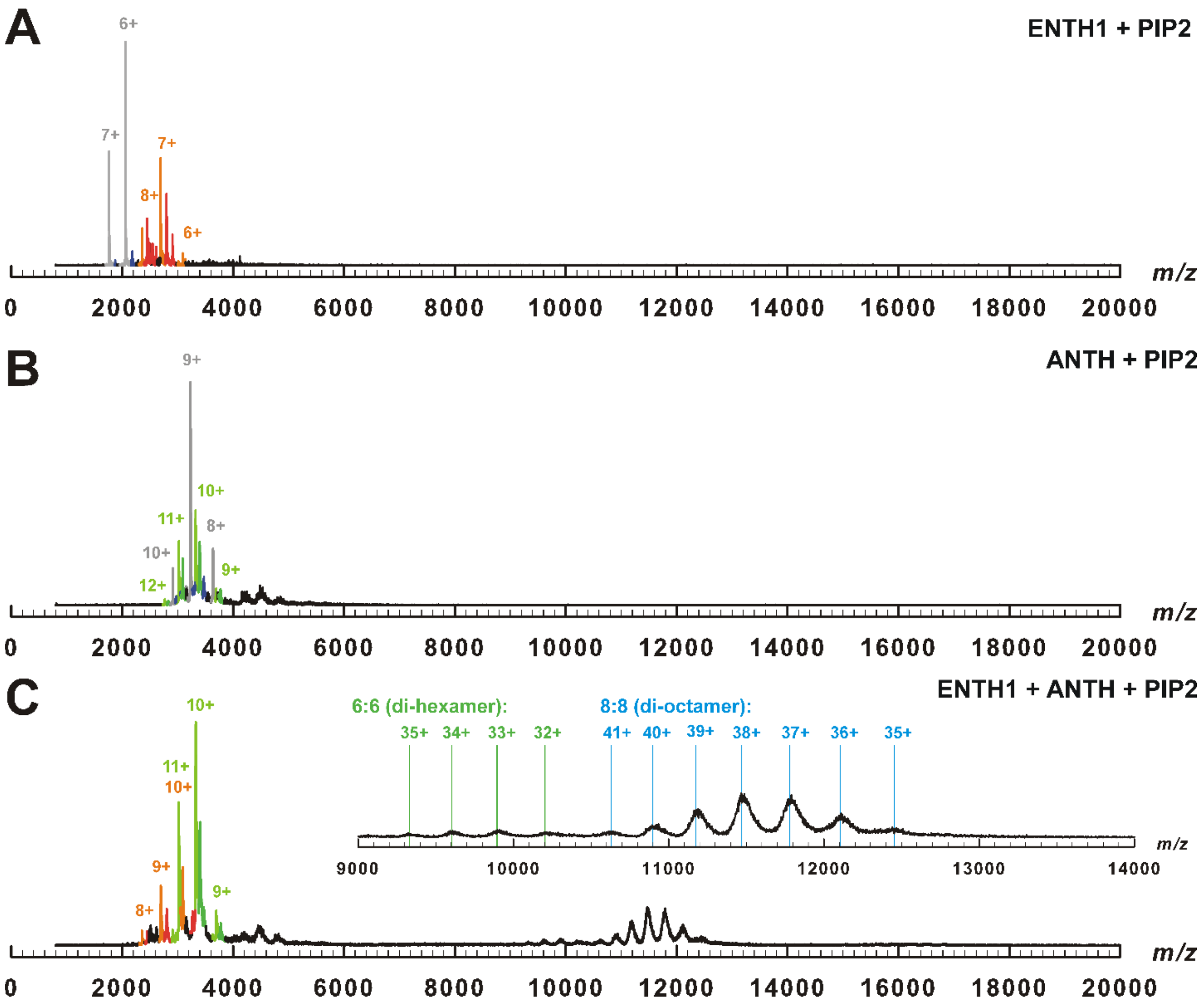

Figure S2Ligand binding and complex formation of yeast ENTH1 and ANTH. Time-of Flight (ToF) mass spectra ofyeast ENTH1 (**A**), yeast ANTH (**B**) and a 1:1 mixture of both (**C**), all in the presence of 6x PIP2 excess. Grey peaks denote cytochrome C (**A**) and carbonic anhydrase (**B**) used as reference proteins for unspecific PIP2. Grey peaks denote cytochrome C (**A**) and carbonic anhydrase (**B**) used as reference proteins for unspecific PIP2 clustering (blue peaks) during the ESI process [4]. Light and dark green as well as orange and red peaks specify adaptor proteins in free and lipid-bound state, respectively.

The *inset* shows an enlarged upper part of the spectrum with blue and red lines indicating the theoretical peak positions of the di-hexamer (green) and of the di-octamer (cyan) complexes with 19 and 26 PIP2 ligands, respectively. Data taken from [2].

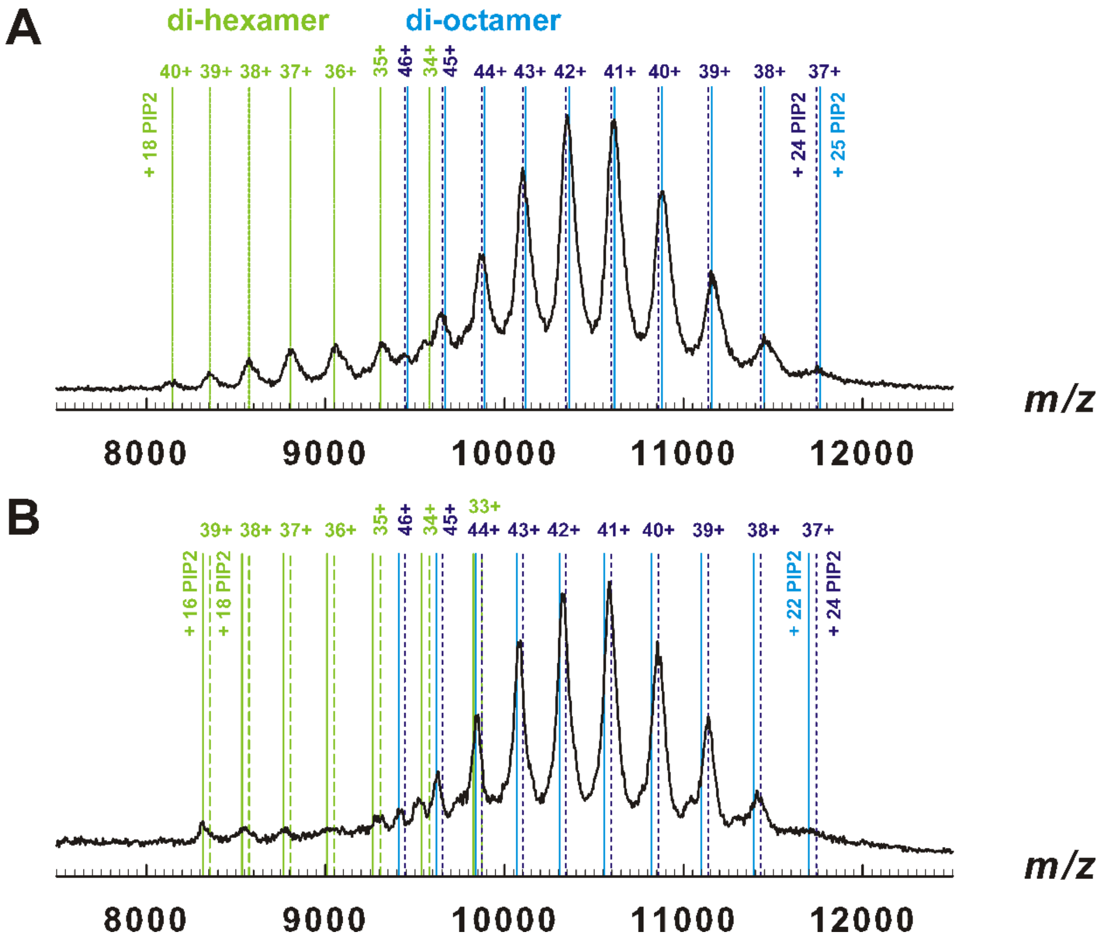

Figure S3 Part of native mass spectra showingcomplex formation of yeast ENTH1-ANTH di-hexa- and di-octamers. Mass spectra of yeast ENTH1 and ANTH were recorded in a Q-ToF1 mass spectrometer modified for native MS after variedsample preparations. **A**: ENTH1 and ANTH were first buffer-exchanged and then assembled with an excess of PIP2 (5.5 µM, 8 µM, and 30 µM, respectively) **B**: the complex was first assembled (same concentrations as above) and then buffer-exchanged, *i.e.* the excess PIP2 was removed from the mixture. Measurements were conducted identically (1.35 kV, 150 V, and 75 V capillary, cone and collision cell voltages, respectively). The spectrum shown in **B**was taken from [3].

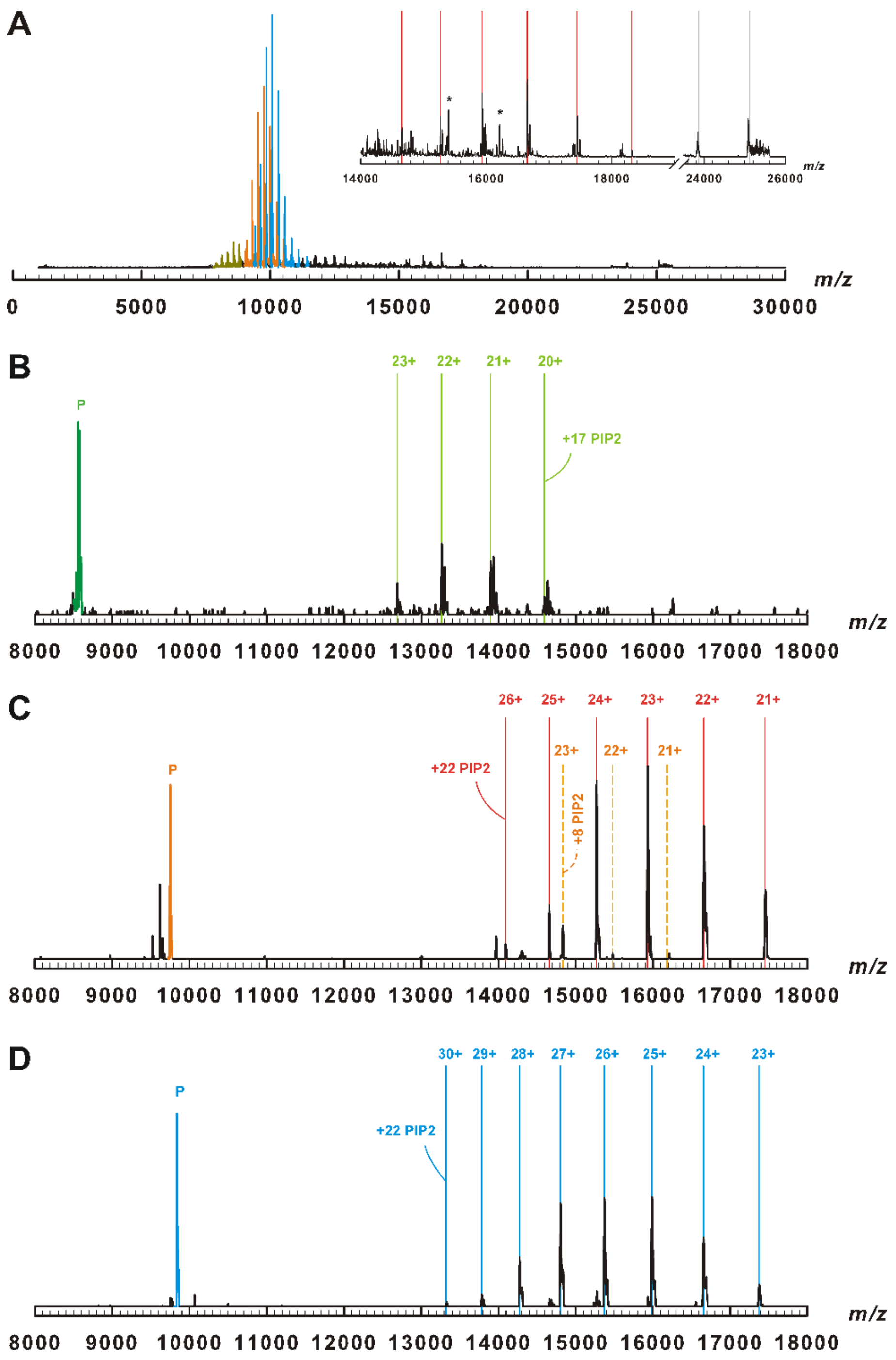

Figure S4Higher order complex formation of yeast ENTH1 and ANTH. A: Mass spectrum of yeast ENTH1 and ANTH assembling into di-hexamers, pentadecamers (8:7) and di-octamers (green, orange and cyan peaks, respectively). The inset shows middle and upper parts of the spectrum enlarged with theoretical peak positions of a putative tetradecamer (8:6) with 22 PIP2 ligands and of an unidentified species of ≈476.75 kDa specified by red and grey lines, respectively. The latter may resemble aheptadecamer (8:9). Asterisks denote a further unidentified species (MW ≈309 kDa). **B-D**: Fragment ion mass spectra of the di-hexamer (**B**), the pentadecamer (8:7) (**C**), and of the di-octamer (**D**). Vertical lines denote the theoretical *m/z* of undecameric (6:5) (**B**), tetradecameric (8:6) (**C**), and pentadecameric (8:7) (**D**) stripped complexes, each carrying the indicated number of PIP2 ligands. See main section, Fig. 1 for close-ups.

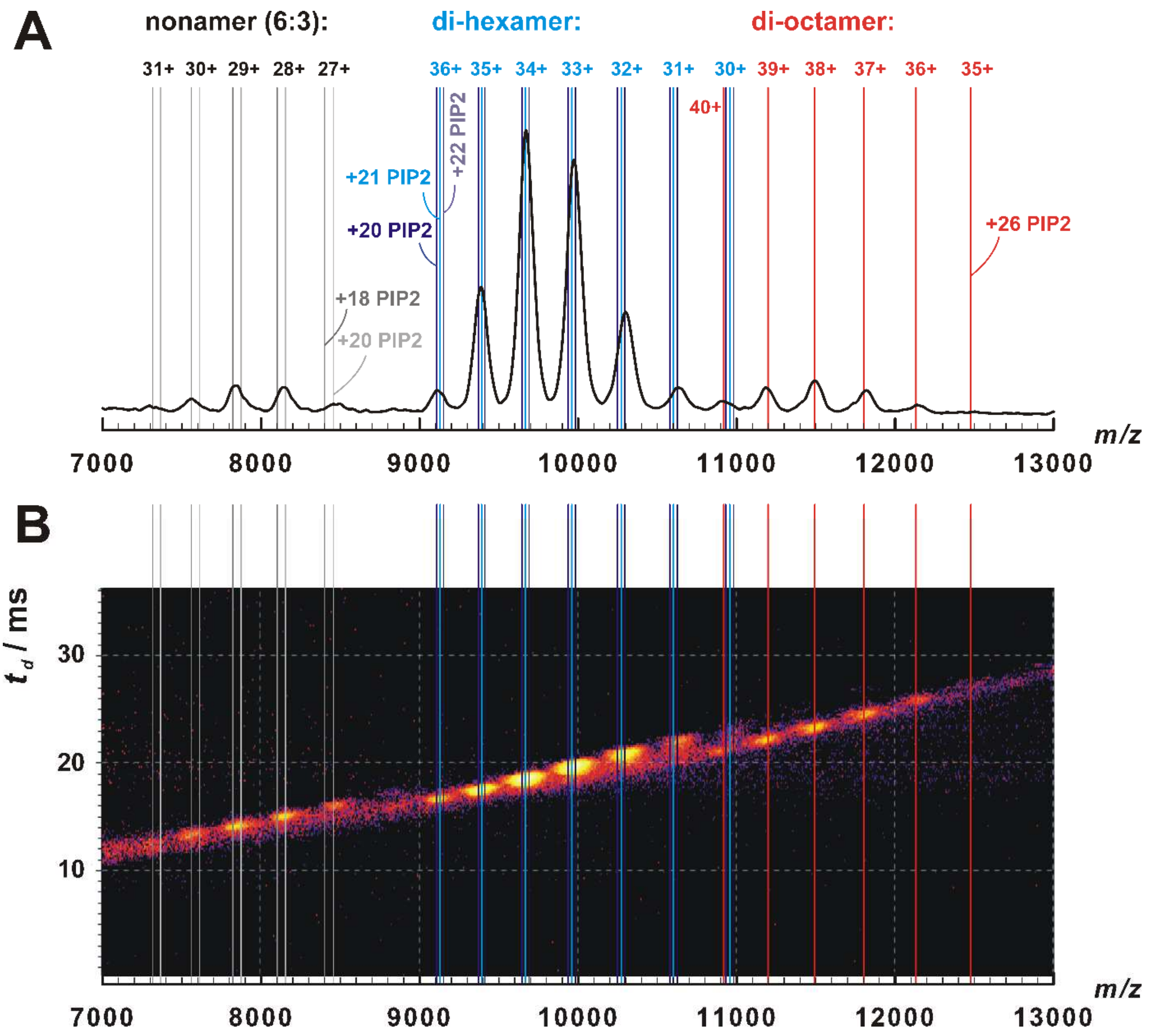

Figure S5Higher order complexes of yeast ENTH2 and ANTH.A: Precursor ion mass spectrum of yeast ENTH2 and ANTH assembling into nonamers (6:3), di-hexamers and di-octamers (from left to right). **B:** Heat map combining MS (horizontal) and ion mobility (vertical) dimensions. The intensity scale is logarithmic. Vertical lines denote theoretical *m/z* of nonamers (6:3), di-hexamers and di-octamers, carrying the indicated number of PIP2 ligands, respectively.

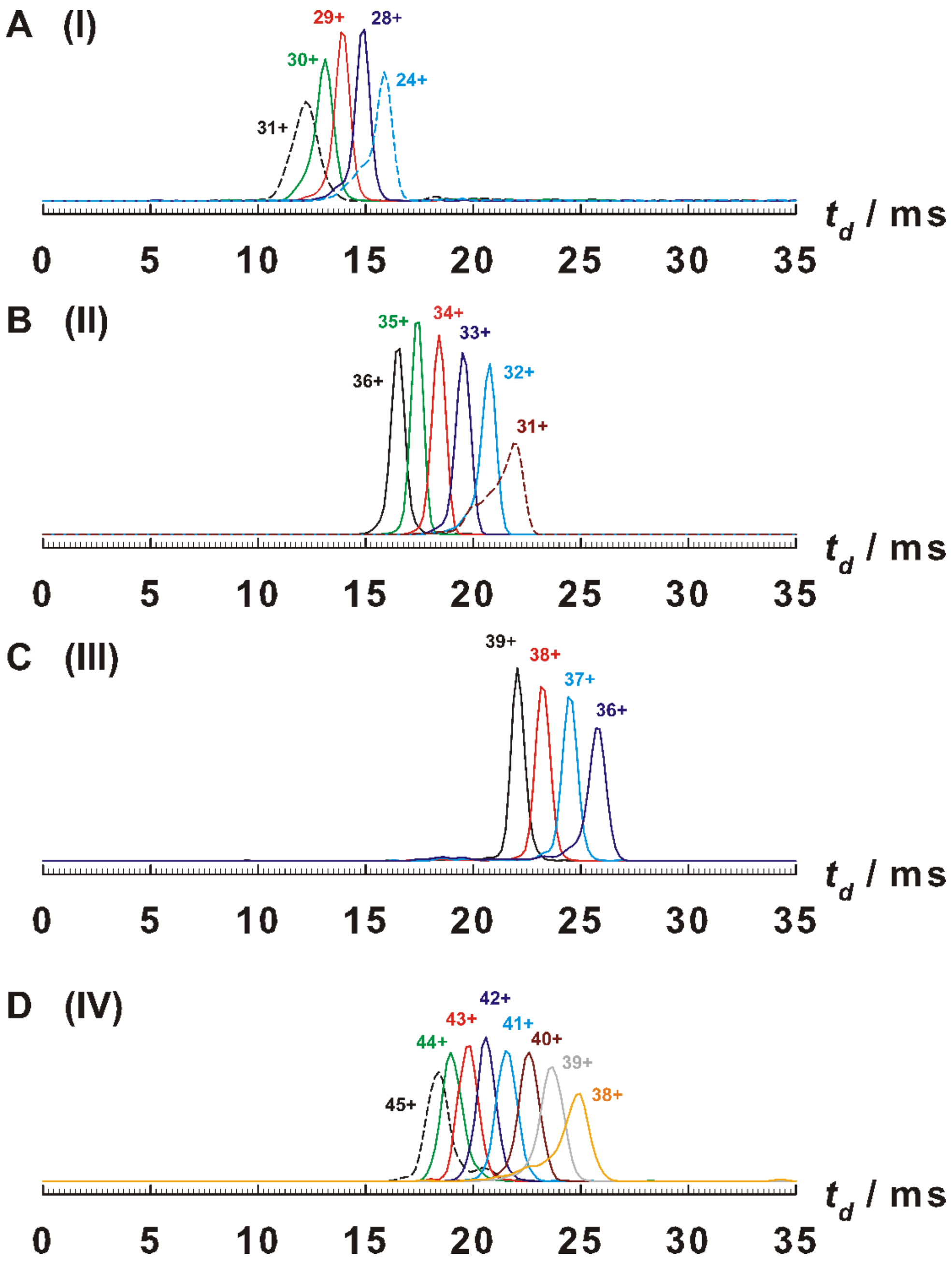

Figure S6Arrival time distributions (ATDs) of yeast ENTH-ANTH complexes. ATDs representing charge states 24+ to 31+ of the ENTH2-ANTH nonamer (6:3) (**A**), 31+ to 36+ of the ENTH2-ANTH di-hexamer (**B**), 24+ to 31+ of the ENTH2-ANTH di-octamer, 36+ to 38+ of the ENTH2-ANTH di-octamer (**C**), and 38+ to 45+ of the ENTH1-ANTH di-octamer (**D**) were extracted from spectra shown in Fig. S4 and Fig. 2 (main section), smoothed and normalized to the total intensity. Roman numbering refers to Tables 1 and 2 in the main section, dashed lines represent ATDs **not**employed for $^{TW}CCS_{N2}$ calculation.

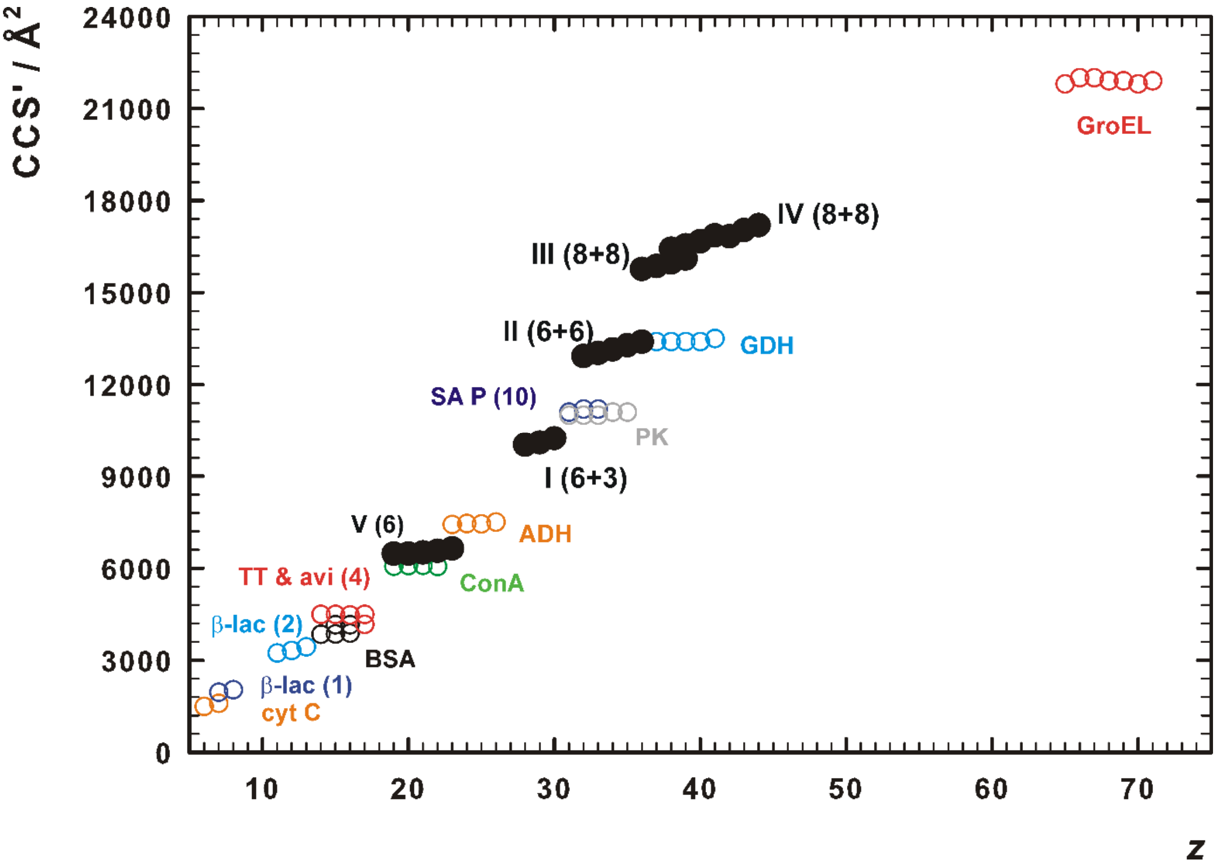

Figure S7 Charge state dependence of individual collision cross sections (CCS'). Calculated CCS' of yeast nonameric (6:3) (I) di-hexameric (II) and di-octameric (III) ENTH2-ANTH or ENTH1-ANTH (IV) complexes as well as of the human ENTH hexamer (V) are plotted versus the corresponding charge state and in comparison with similar data of the indicated reference proteins. Data of cytochrome C (cyt C), β-lactoglobulin (β-lac) transthyretin (TT), avidin (avi), serum albumin (BSA), concanavalin A (ConA), alcohol dehydrogenase (ADH), serum amyloid P (SA P), pyruvate kinase (PK), glutamate dehydrogenase (GDH) and GroEL were taken from [5]. If deemed necessary, oligomerization status indicated by numbers in in brackets, Roman numbering refers to Tables 1 and 2 in the main section.

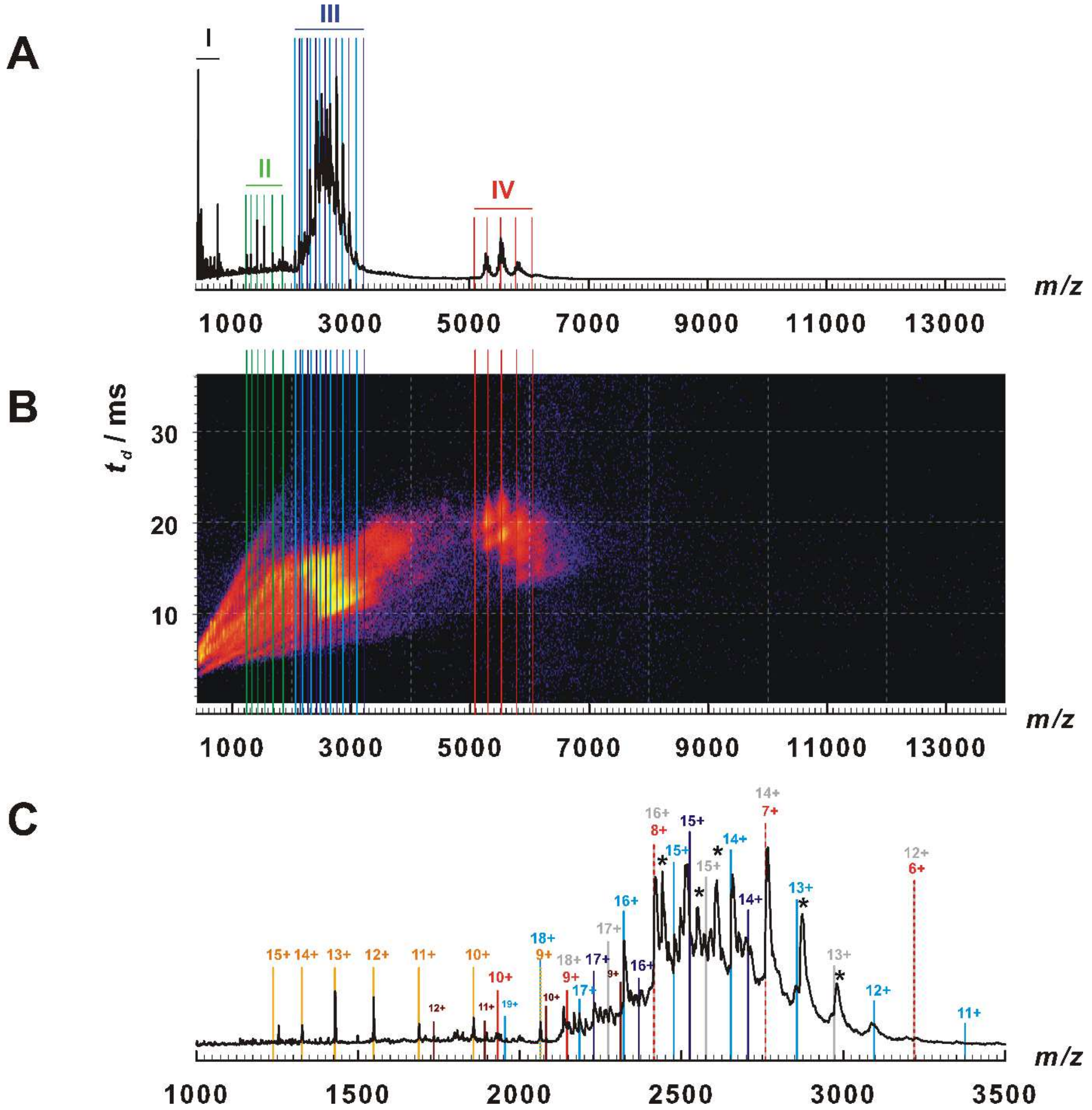

Figure S8 Native Ion Mobility Mass Spectrum of human ENTH in different association states. A: Precursor ion mass spectrum comprising free PIP2 with fragments thereof and sodium adducts (I), monomeric free ENTH (II), a mixture of PIP2-bound monomeric and dimeric (both, free and lipid-bound) ENTH (III) and hexameric ENTH (IV). Vertical lines indicate theoretical *m/z* free monomeric (green), free (cyan) or doubly ligand-bound (blue), and hexameric (red) ENTH. **B:** Heat map combining MS (horizontal) and ion mobility (vertical axis) dimensions. The intensity scales is logarithmic. **C:** Enlarged lower part of the spectrum shown in **A**, with the theoretical *m/z* of free, singly or doubly PIP2-bound monomericENTH denoted by orange, red, and dark red lines, respectively, as well as those of free, singly or doubly PIP2-bound ENTH dimers indicated by cyan, dark blue, and grey ones. *: unidentified species. See **Figure S9B, upper panel** for a similar close-up of region IV.

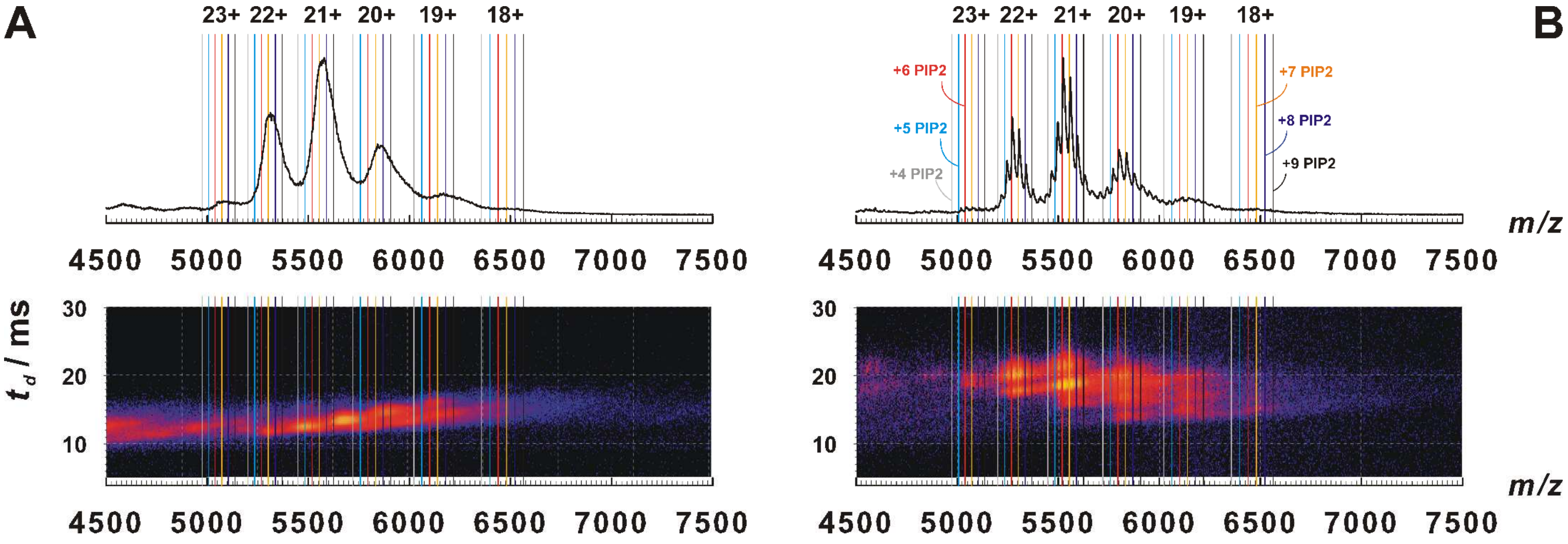

Figure S9 Human ENTH hexamer at different conditions. Mass spectra acquired with 25 V (**A**) and 75 V (**B**) in the trap cell, respectively. **Upper panels:** Parts of the precursor ion mass spectra comprising the hexameric complexes. **Lower panels:** Heat maps combining MS (horizontal) and ion mobility (vertical axis) dimensions. Intensity scales are logarithmic. Vertical lines denote theoretical *m/z* of hexameric ENTH with four to nine PIP2 ligands bound, as indicated.

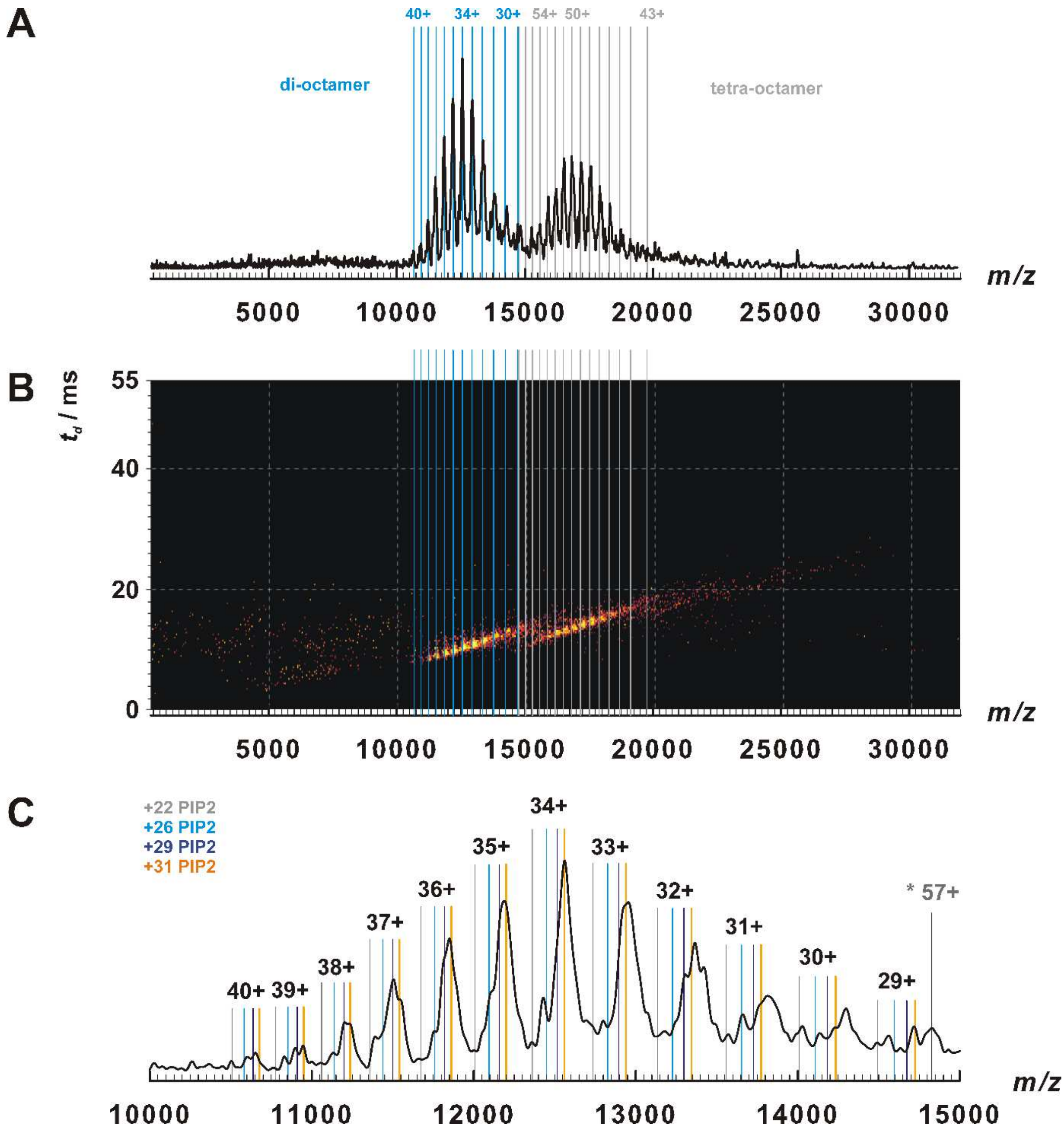

Figure S10Native Ion Mobility Mass Spectrum of yeast ENTH1-ANTH complexes at 30 V SID voltage. A: Precursor ion mass spectrum with MS profile applied showing di-octameric ENTH1-ANTH complexes and dimers thereof (tetra-octamers) .**B:** Heat map combining MS (horizontal) and ion mobility (vertical axis) dimensions. The intensity scale is logarithmic. Vertical lines mark theoretical *m/z* of di-octamers and tetra-octamers with 31 and 45 ligands bound, respectively. **C:** Enlarged part of the spectrum shown in **A**, vertical lines denoting the theoretical *m/z* of di-octamers binding 22-31 PIP2 molecules as indicated. *: The vertical grey line marks the beginning of the tetra-octamer peak series (see **A**).

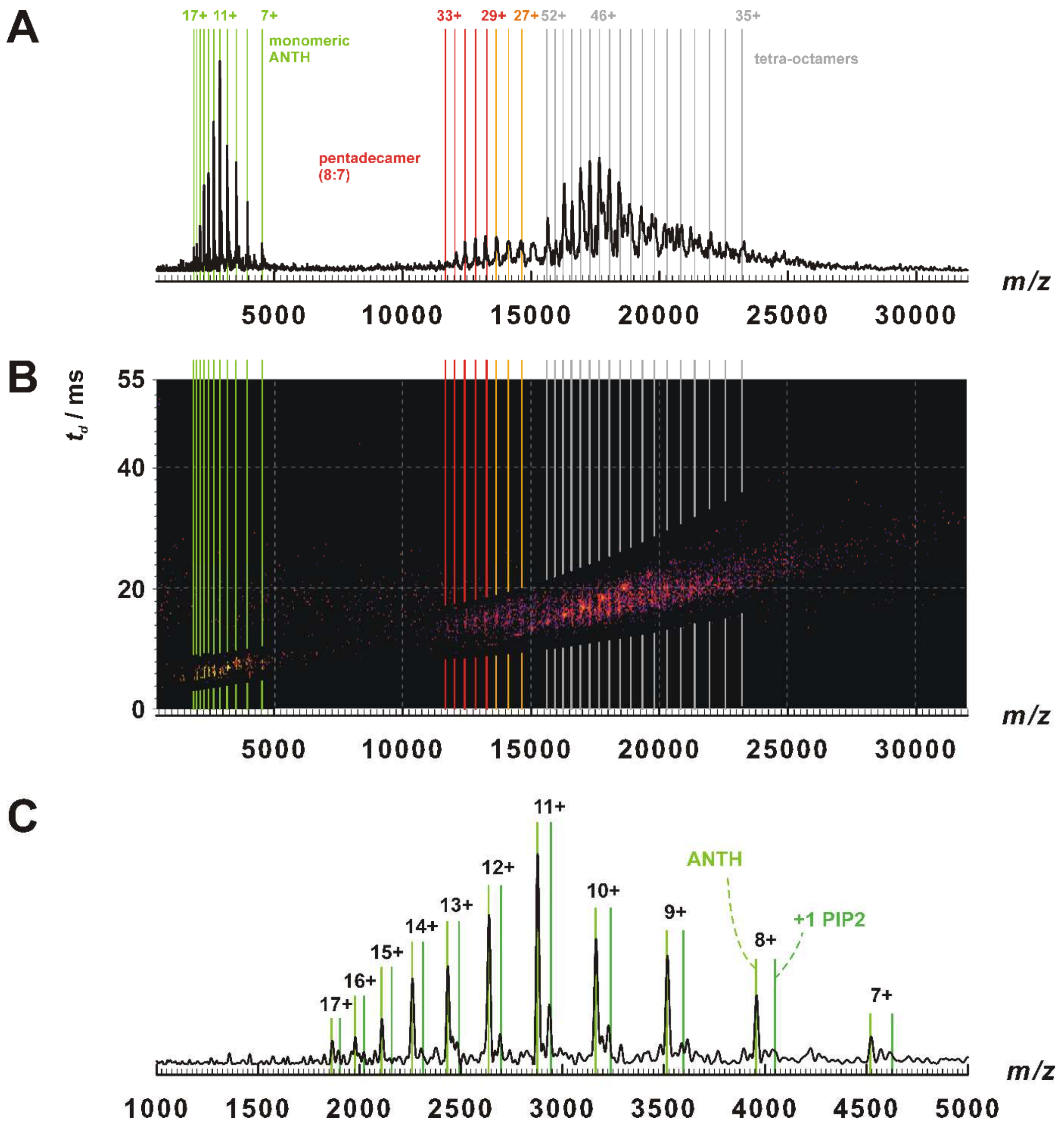

Figure S11Native Ion Mobility Mass Spectrum of yeast ENTH1-ANTH complexes at 90 V SID voltage. A: Precursor ion mass spectrum with MS profile applied showing dissociation products of di-octameric ENTH1-ANTH complexes. **B:** Heat map combining MS (horizontal) and ion mobility (vertical axis) dimensions. The intensity scales is logarithmic. Vertical lines indicate theoretical *m/z* of free ANTH, pentadecamers (8:7)with 17 (red) or 31 (orange) PIP2 ligands bound as well as of the tetra-octamers. **C:** Enlarged part of the spectrum shown in **A**, with light green and green vertical lines denoting the theoretical *m/z* of free and singly ligand-bound ANTH, respectively.

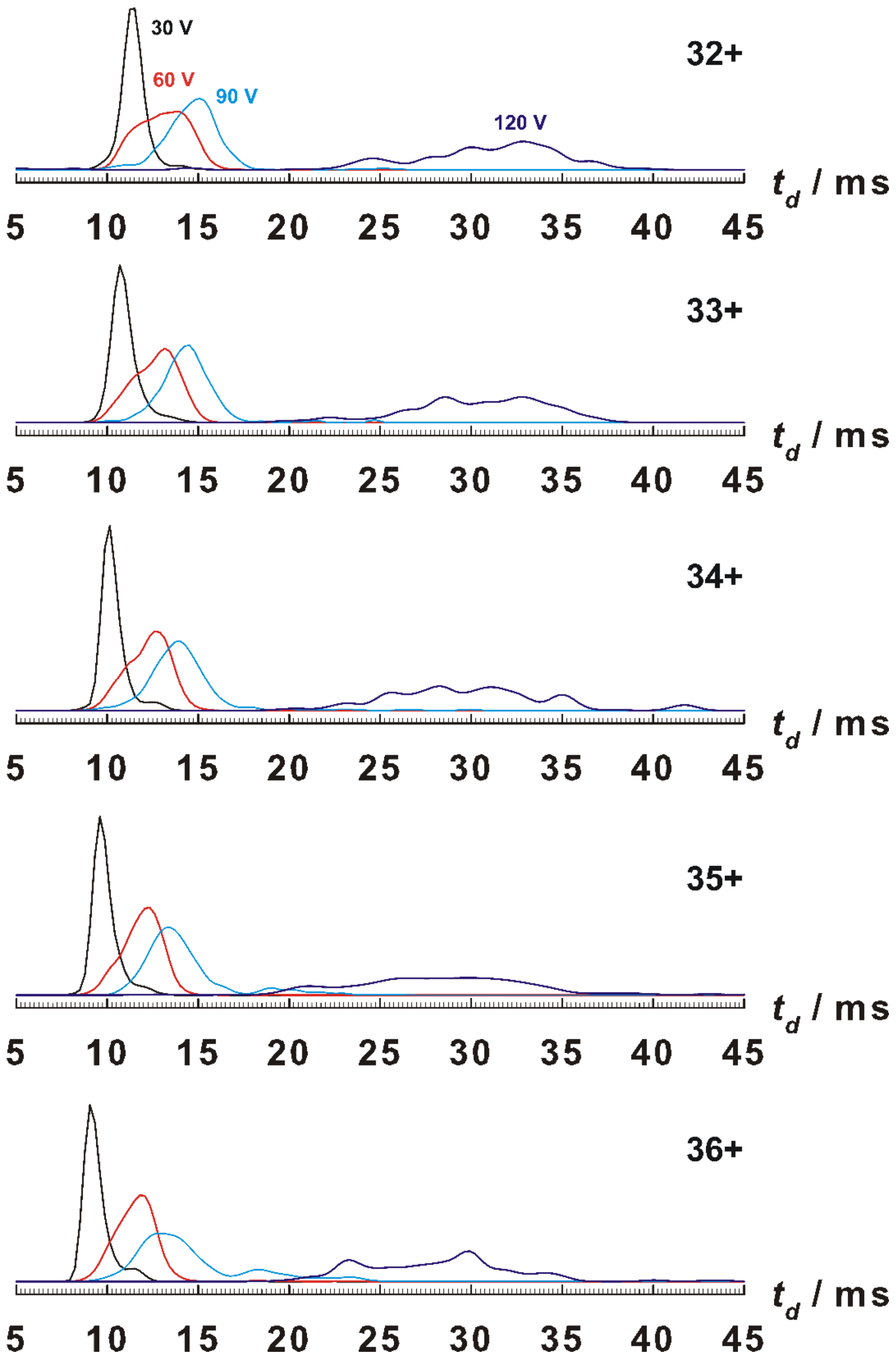

Figure S 12 Arrival time distributions (ATDs) of yeast ENTH1-ANTH di-octamers at increasing SID voltages.ATDs representing charge states 32+ to 36+ (from top down) of the ENTH1-ANTH di-octamer carrying ≈30 PIP2 ligands were extracted from spectra recorded with SID voltages of 30 V, 60 V, 90 V, or 120 V as indicated in the top panel, smoothed and normalized to the total intensity.

References:

1. Messa, M., et al., *Epsin deficiency impairs endocytosis by stalling the actin-dependent invagination of endocytic clathrin-coated pits.* Elife, 2014. **3**: p. e03311.
2. Garcia-Alai, M.M., et al., *Epsin and Sla2 form assemblies through phospholipid interfaces.* Nat Commun, 2018. **9**(1): p. 328.
3. Skruzny, M., et al., *An organized co-assembly of clathrin adaptors is essential for endocytosis.* Dev Cell, 2015. **33**(2): p. 150-62.
4. Sun, J., et al., *Method for distinguishing specific from nonspecific protein-ligand complexes in nanoelectrospray ionization mass spectrometry.* Anal Chem, 2006. **78**(9): p. 3010-8.
5. Bush, M.F., et al., *Collision cross sections of proteins and their complexes: a calibration framework and database for gas-phase structural biology.* Anal Chem, 2010. **82**(22): p. 9557-65.
